# Electrophysiology consumables procurement in Europe: implications for access, innovation and value-based care

**DOI:** 10.1093/europace/euag039

**Published:** 2026-03-10

**Authors:** Lucía Osoro, Nikola Kozhuharov, Runa Landen, Elena Arbelo, Martin Martinek, Christophe Leclerq, Laurent Fauchier, Jean-Claude Deharo, Serge Boveda, Philipp Sommer, Michiel Rienstra, Piotr Szymanski, Michal Farkowski, Francisco Costa, Diana Tint, Stefan Simovic, Krasimir Dzhinsov, Francisco Leyva, Giuseppe Boriani, Josep Figueras, Zenichi Ihara, Jose Luis Merino Llorens, Haran Burri, Helmut Pürerfellner, Rubén Casado-Arroyo

**Affiliations:** Department of Cardiology, H.U.B.-Hôpital Erasme, Université Libre de Bruxelles, Av. Franklin D. Roosevelt 50, Brussels 1050, Belgium; EHRA Advocacy, Quality Improvement, and Health Economics Committee (European Heart Rhythm Association) 2025 route des Colles, Les Templiers, 06903 Sophia Antipolis, France; Centro Universitario HM Hospitales de Ciencias de la Salud (CUHMED), Universidad Camilo José Cela, C. Castillo de Alarcon 49, Urb. Villafranca del Castillo, 28692, Villanueva de la Cañada, Spain; EHRA Advocacy, Quality Improvement, and Health Economics Committee (European Heart Rhythm Association) 2025 route des Colles, Les Templiers, 06903 Sophia Antipolis, France; Department of Cardiology, University Hospital Bern—Inselspital, Freiburgstrasse 20, Bern 3010, Switzerland; EHRA Advocacy, Quality Improvement, and Health Economics Committee (European Heart Rhythm Association) 2025 route des Colles, Les Templiers, 06903 Sophia Antipolis, France; Department of Cardiology, Institute of Medicine—Sahlgrenska Academy—University of Gothenburg, Gothenburg, Sweden; EHRA Advocacy, Quality Improvement, and Health Economics Committee (European Heart Rhythm Association) 2025 route des Colles, Les Templiers, 06903 Sophia Antipolis, France; Department of Cardiology, Hospital Clinic, Barcelona, Spain; Department of Cardiology, Ordensklinikum Linz Elisabethinen, Linz, Austria; Department of Cardiology, CHU Rennes—Hôpital Pontchaillou, Rennes, France; Department of Cardiology, Hôpital Trousseau, CHRU de Tours, France; Assistance Publique − Hôpitaux de Marseille, Centre Hospitalier Universitaire La Timone, Service de Cardiologie, Marseille, France; Department of Cardiology, Aix Marseille Université, C2VN, Marseille, France; Department of Cardiology, Clinique Pasteur, Toulouse, France; Heart and Diabetes Center North Rhine-Westphalia, University Clinic of Bochum, Bad Oeynhausen, Germany; Department of Cardiology, University of Groningen, University Medical Centre Groningen, Groningen, The Netherlands; Centre of Postgraduate Medical Education, Warsaw, Poland; Cardiology, Ministry of Interior and Administration National Medical Institute, Warsaw, Poland; Department of Cardiology, Hospital Santa Cruz, Lisbon, Portugal; Department of Cardiology, Transilvania University of Brasov, Brasov, Romania; Department of Internal Medicine, Faculty of Medical Sciences, University of Kragujevac, Kragujevac Serbia; Clinic for Cardiology, University Clinical Centre Kragujevac, Kragujevac, Serbia; Department of Cardiology, University Hospital ‘Sveti Georgi’, Plovdiv, Bulgaria; Department of Cardiology, Aston University, Birmingham, United Kingdom of Great Britain & Northern Ireland; Department of Cardiology Policlinico di Modena, Italy; European Health Observatory, on Health Systems and Policies, Brussels, Belgium; Department of Health Economics and Reimbursement, Abbott, Zaventem, Belgium; Robotic Arrhythmia and Electrophysiology Unit, La Paz University Hospital, Madrid, Spain; Cardiology Department, University Hospital of Geneva, Geneva, Switzerland; Department of Cardiology, Ordensklinikum Linz Elisabethinen, Linz, Austria; Department of Cardiology, H.U.B.-Hôpital Erasme, Université Libre de Bruxelles, Av. Franklin D. Roosevelt 50, Brussels 1050, Belgium; EHRA Advocacy, Quality Improvement, and Health Economics Committee (European Heart Rhythm Association) 2025 route des Colles, Les Templiers, 06903 Sophia Antipolis, France

**Keywords:** Electrophysiology, Public procurement, Catheters, Value-based healthcare, Innovation, Health systems, Europe

## Abstract

**Aims:**

To examine and compare public procurement systems for electrophysiology (EP) consumables across 21 European countries, focusing on governance level, evaluation methods, clinician involvement, reimbursement variability, access to innovation, and sustainability integration.

**Methods and results:**

A qualitative, exploratory design was employed using 22 semi-structured interviews with EP clinicians, procurement specialists, and health system stakeholders across 21 countries. Interview transcripts and summaries were thematically coded by two independent researchers using a structured six-domain framework. Substantial heterogeneity in procurement practices was identified. Hospital-level procurement predominates in 43% of countries, while 33% use regional-level tenders; a minority operate national-level frameworks. Evaluation methods vary, with several countries using price-driven criteria, while others apply mixed or clinically weighted models. Clinician involvement is high or moderate in two-thirds of countries but often informal or lacking governance structure. Reimbursement for EP procedures varies widely in scope and transparency, with bundled and global budget models affecting innovation uptake. Innovation access remains uneven: countries such as Austria, the Netherlands, and France use innovation funds or dedicated pathways, while others rely on centralized approvals or re-tendering. Sustainability criteria are rarely formalized in procurement decisions, despite growing awareness of environmental impact.

**Conclusion:**

European procurement systems for EP consumables differ markedly in structure, evaluation practices, and alignment with clinical and innovation priorities. Integrating clinician input, adopting value-based frameworks, and embedding sustainability metrics could enhance procurement outcomes and patient care. Harmonized guidance from EHRA and EU-level stakeholders may support more equitable and innovation-friendly procurement strategies.

## Graphical Abstract

**Graphical Abstract:**
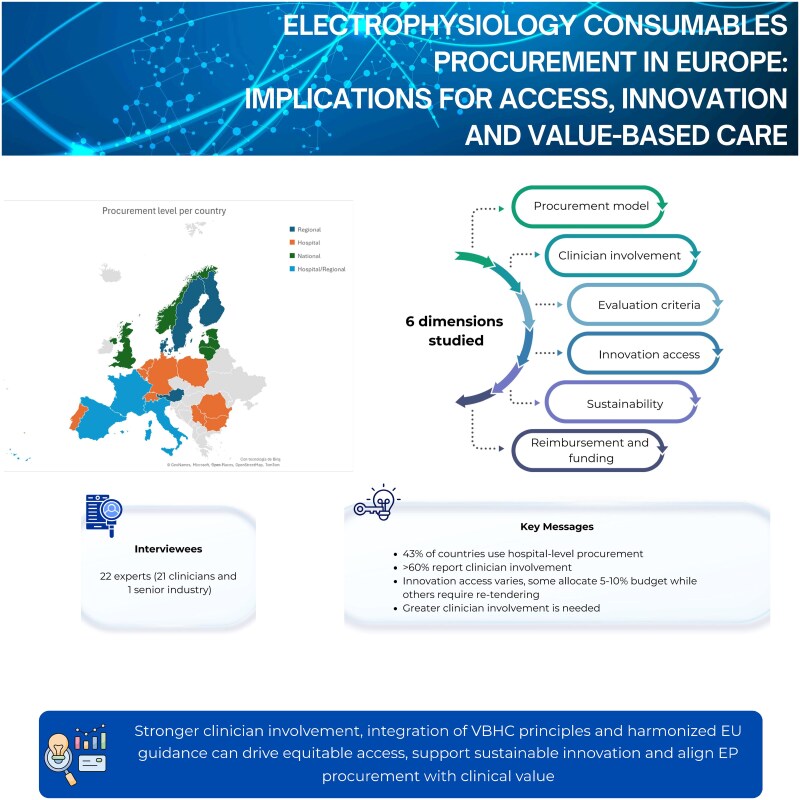

## Table of contents

Introduction
Methods
 Study design
 Participant selection
 Data collection
 Data analysis
 Reimbursement data
 Limitations and bias mitigation
Results
 Procurement models
 Evaluation methods: cost vs. clinical criteria
 Clinician involvement
 Reimbursement variability across EP procedures
 Access to innovation
 Funding and rebate mechanisms
Discussion
 Procurement models and clinical integration
 Clinician involvement: a determinant of value alignment
 Evaluation criteria and value-based procurement
 Role of health technology assessment in value-based procurement
 Access to innovation and funding mechanisms
 Environmental sustainability as an emerging criterion
Conclusions
Supplementary material
Conflict of interest
Data availability
Funding
References

What’s new?

- This is the first cross-country analysis of procurement processes for electrophysiology consumables in 21 European countries.
- The study identifies substantial variability in procurement models, clinician involvement, access to innovation, evaluation methods and sustainability.
- Over 60% of countries report clinician involvement in procurement; 43% rely on hospital-level purchasing.
- Access to innovation is fragmented: Some countries allocate up to 10% for novel technologies, while others require full re-tendering.
- Greater clinician involvement, adherence to value-based healthcare criteria, and EU-level policy coordination are key to improving access and sustainability.
- Environmental sustainability is rarely considered but offers a growing opportunity for procurement reform.

## Introduction

Catheters, sheaths, and electroanatomical mapping systems are essential for the safe and effective performance of electrophysiology (EP) procedures. These consumables enable contemporary workflows such as zero-fluoroscopy ablation, reducing radiation exposure for both patients and healthcare professionals, while high-quality signal acquisition and real-time interpretation remain critical for identifying arrhythmogenic substrates and achieving durable procedural outcomes.^1^

Technological advances have progressively improved diagnostic accuracy and ablation efficacy in EP. High-density multipolar mapping catheters, visualizable steerable sheaths, and robotic or magnetic navigation systems have enhanced anatomical reconstruction, lesion delivery, and procedural precision. In parallel, the availability of multiple ablation modalities, including radiofrequency catheters with contact-force sensing, cryoballoon technology, pulsed field ablation (PFA), and laser-based systems, has expanded treatment options for atrial and ventricular arrhythmias, supporting more individualized therapeutic strategies, with the triple objective of reducing procedure time, risk of complications, and improving outcomes.^1^

Although the integration of advanced consumables has increased procedural costs, their clinical and economic value is well documented. Improvements in lesion durability, procedural efficiency, and arrhythmia-free survival reduce the need for repeat interventions and may translate into long-term cost savings. As such, EP consumables represent not only technical tools but also key contributors to high-value care delivery.^1^

At the same time, their widespread adoption raises challenges related to environmental sustainability and equitable access. The reliance on single-use catheters and accessories generates substantial medical waste. EHRA surveys have shown that most EP consumables are discarded after one use, despite European regulatory frameworks permitting controlled reprocessing under defined conditions. This highlights a disconnect between sustainability objectives and current procurement and operational practices. Incorporating environmental criteria, such as lifecycle impact, reusability, and packaging waste, into procurement decisions could help align innovation with ecological responsibility.^2,3^

Despite these developments, procurement practices for EP consumables remain highly heterogeneous across Europe. Limited data exist on how health systems balance price and clinical value, the extent of clinician involvement in procurement governance, and whether innovation and sustainability are formally supported. Addressing these gaps is necessary to better align procurement policies with evolving EP practice and broader health system objectives.^4,5^

This study aims to map and compare public procurement systems for EP consumables across 21 European countries. Using a structured interview-based methodology, we examine governance models, evaluation criteria, clinician involvement, access to innovation, reimbursement, funding mechanisms, and sustainability. Based on these findings, we propose recommendations to support value-based, equitable, and sustainable procurement strategies in EP.

## Methods

### Study design

This study employed a qualitative, exploratory design based on semi-structured interviews to examine public procurement processes for EP consumables and related technologies used in arrhythmia management across Europe. The qualitative approach was selected to capture system-level differences in procurement governance, evaluation criteria, clinician involvement, access to innovation, reimbursement mechanisms, and sustainability practices across diverse healthcare settings.

### Participant selection

Participants were purposively selected to ensure broad geographic representation and direct experience with EP procurement processes. The study included 22 participants across 21 European countries, comprising 21 clinicians and 1 representative from the medical technology industry.

All clinician participants were practicing cardiologists with expertise in EP and were affiliated with the European Society of Cardiology or their respective national cardiac societies. Clinicians were selected based on their direct involvement in procurement activities within their institutions, including participation in device specification, evaluation or purchasing decisions, as well as their familiarity with local procurement, reimbursement, and decision-making frameworks related to EP consumables.

One participant represented the medical technology industry and covered the Baltic and Nordic regions. Industry participation was intentionally limited to a single interviewee to avoid disproportionate influence on qualitative findings while allowing triangulation of clinician-reported procurement practices with a market-level perspective.

### Data collection

Interviews were conducted online via videoconferencing between 19 September 2024 and 27 May 2025 using a structured interview guide developed from a preliminary literature review and expert consultation. Interviews lasted between 17 and 27 min and were conducted in English, French, or Spanish, according to participant preference, to facilitate clear discussion of complex procurement and reimbursement processes.

All interviews were audio- or video-recorded with participants’ informed verbal consent. A written summary of each interview was prepared and returned to the participant for verification and clarification prior to analysis. Ethical approval was not required, as no patient data were collected and participants contributed in a professional and institutional capacity.

### Data analysis

Interview summaries and transcripts were analysed using an inductive thematic analysis approach. Initial coding was performed independently by two researchers to identify recurring concepts and patterns. Codes were subsequently reviewed, discussed, and refined to ensure consistency and consensus.

Emergent themes were organized into six analytical domains: procurement governance level, evaluation criteria, clinician involvement, access to innovation, reimbursement and funding mechanisms, and sustainability integration. This framework enabled structured cross-country comparison while allowing additional context-specific themes to emerge. The NVivo software (QSR International) was used to support data management and thematic organization.

### Reimbursement data

Information on reimbursement structures was obtained through interviews and supplemented with publicly available national sources where accessible. Given substantial variation in reimbursement models, contracting arrangements, and procedural complexity within and between countries, precise monetary values were not used in the primary analysis. Instead, reimbursement was categorized using indicative ranges and structural characteristics to emphasize system design rather than direct price comparison and to avoid misinterpretation of locally negotiated tariffs.

### Limitations and bias mitigation

Several limitations should be acknowledged when interpreting these findings. First, although purposive sampling enabled the inclusion of participants with direct involvement in EP procurement processes, the qualitative design does not capture the full complexity of procurement systems within each country. In several countries, findings are based on a single interviewee, which may limit the representation of regional or institutional variability, particularly in decentralized healthcare systems.

Secondly, procurement and reimbursement practices may vary substantially within countries, especially between public and private hospitals or across regions. As a result, the information reported may reflect institutional rather than fully national practices. These sources of intra-country heterogeneity should be considered when interpreting country-level comparisons.

Thirdly, the predominance of clinicians among participants may have emphasized procedural and patient-centred perspectives, while administrative or policy-level viewpoints may be underrepresented. This was partially mitigated by the inclusion of one medical technology industry representative, providing contextual market-level insights without disproportionately influencing the qualitative analysis.

To enhance data credibility and reduce bias, interviews were conducted using a standardized guide and focused on participants’ areas of direct responsibility and experience. Where procurement or reimbursement practices extended beyond an individual’s scope, this was explicitly acknowledged and reflected in the analysis. In addition, interview summaries were returned to participants for verification and clarification prior to coding, reducing the risk of misinterpretation.

Interviews were conducted in English, French, or Spanish according to participant preference to facilitate accurate discussion of complex technical, regulatory, and policy-related concepts and to minimize language-related response bias.

Finally, reimbursement data were synthesized using indicative ranges and structural categories rather than precise monetary values. This approach was chosen to account for variability in local contracting arrangements, bundled payments, and negotiated rebates and to avoid misinterpretation of centre-specific tariffs. As such, reimbursement figures should be interpreted as illustrative of system design rather than definitive price benchmarks.

## Supplementary Material

euag039_Supplementary_Data

## Figures and Tables

**Figure 1:**
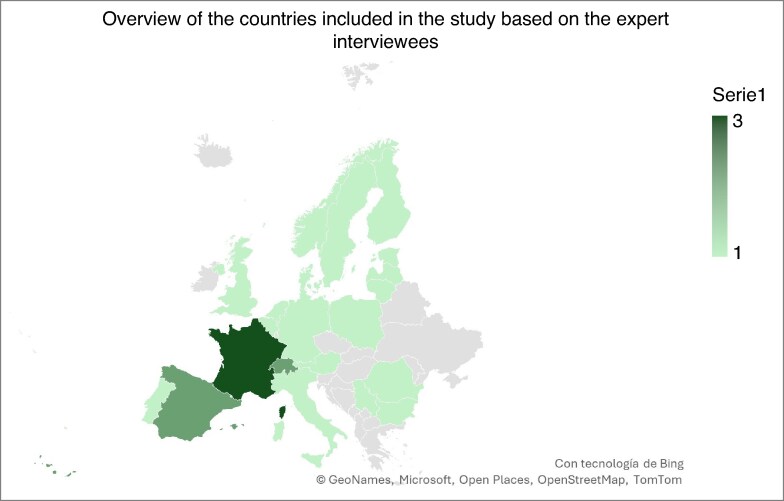
Map showing the number of interviewees per country involved in the study.

**Table 1:** Summary procurement scoring matrix for EP goods

Country	Procurement level	Clinician involvement	Innovation access	Evaluation method	Sustainability integration
AUSTRIA	Hospital level (EP) (1)	High	Innovation dependent on hospital size/budget	Mixed (50/50)	Limited
BELGIUM	Hospital level	Moderate	Limited (via hospital budget)	Mixed (price + quality)	Limited
BULGARIA	National (with hospital-level execution)	Moderate	Funded by NHI, slow uptake	Price-driven	None
DENMARK	Regional	Moderate	Not structured	Mixed	Not defined
ESTONIA	National	Low	Limited	Price-driven	Public pricing (regulated)
FINLAND	Regional	Moderate to high	Included in value model	Lifecycle/VBHC	Risk-sharing contracts
FRANCE	Regional/hospital	High	Local innovation funds	MEAT (up to 85% clinical weight)	Framework agreements
GERMANY	Hospital level	High	Case-by-case (hospital budget)	Variable (hospital-based)	Local-level decisions
ITALY	Regional/national	High	Regional HTA approval	Weighted (e.g. 55% price)	Regional strategy
LATVIA	National	Moderate	Joint Baltic tenders	Price-dominant	Not specified
LITHUANIA	National	Low	New tender required	Fixed-price	Centrally defined volumes
NETHERLANDS	Hospital level	High	10% innovation budget	Hospital-led MEAT	Local (hospital level)
NORWAY	National	Moderate	Annual update index	50/50 MEAT	Central authority
POLAND	Hospital level	High	Hospital innovation funds	Variable	Decentralized
PORTUGAL	Hospital level	Moderate	Package-based approval	Committee-driven	National oversight
ROMANIA	Hospital level	Moderate	Limited	Budget-capped	Defined by hospital
SERBIA	Hospital level	Moderate	Requires insurer pre-approval	Price-dominant	Not specified
SPAIN	Regional/hospital	High	Procedure-based innovation funding	Mixed (framework + clinical)	Hospital-level rebates
SWEDEN	Regional	High	Direct tender flexibility	Points-based scoring	Sustainability pilots
SWITZERLAND	Hospital level	High	Hospital budget approval	Clinical + price balance	Hospital rebates
UK	National (centralized via NHS Supply Chain)	High (in advisory roles)	Case-by-case	Price-led with advisory input	Under development

(1) In Austria, EP consumables are typically procured through hospital-level negotiations without formal tenders. Innovation access varies by hospital capacity and budget.

**Figure 2:**
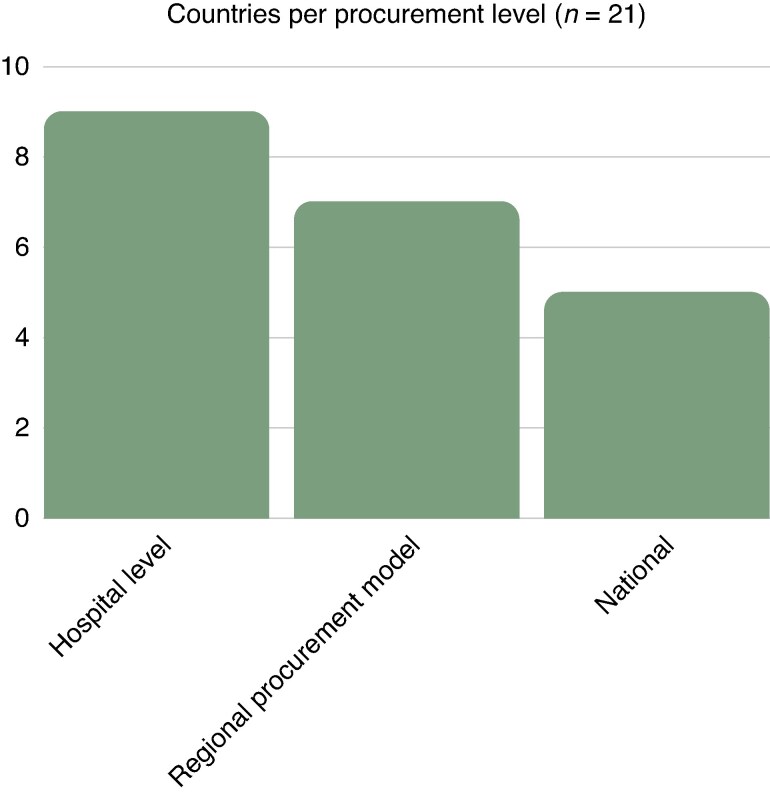
Distribution of procurement models. Countries categorized by dominant level (hospital, regional, or national) based on expert interviews. This figure illustrates the primary procurement governance models for EP consumables reported across 21 European countries. Procurement levels are categorized as hospital level, regional, or national, based on stakeholder interviews. Hospital-based procurement dominates in 43% of countries, while 33% follow regional models and a minority utilize national-level frameworks. This variability reflects differing health system structures and degrees of centralization, with implications for standardization, efficiency, and equity in technology access.

**Figure 3:**
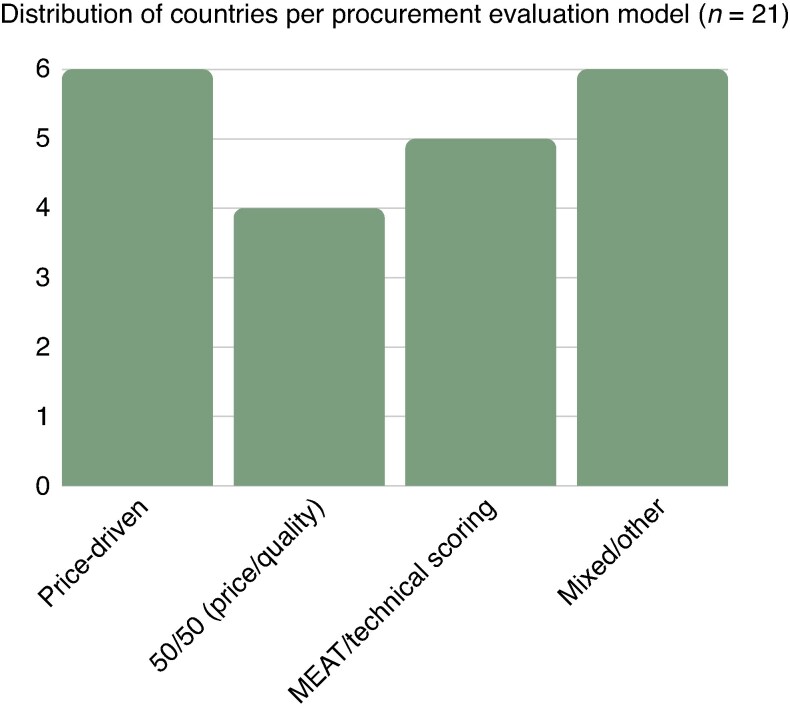
Weight of pricing vs. technical criteria in procurement decisions. This figure displays the predominant evaluation approaches used in public procurement of EP consumables across Europe. Countries are grouped based on the relative weight assigned to price vs. technical/clinical criteria. Models include price-driven, mixed, MEAT, or clinical-led scoring. The figure highlights substantial variation: while some systems prioritize cost minimization, others incorporate clinical value, clinician input, or broader VBHC metrics. This heterogeneity may impact innovation adoption and patient outcomes.

**Table 2:** Summary of EP procedure reimbursement characteristics across Europe

Country	Reimbursement model	Reimbursement level (indicative)	Consumables coverage
Austria	DRG bundled	Medium–High	Implicitly covered
Belgium	Device-on-top	Medium	Explicit
France (Pub)	DRG bundled	Medium	Implicitly covered
France (Priv)	Fee-for-service	High (physician fee separate)	Explicit
Germany	DRG bundled	High	Implicit or pooled rebate
Italy	DRG bundled + regional HTA	Medium	Implicit (varies by region)
Netherlands	DRG bundled	Medium	Pooled at hospital level
Poland	DRG bundled	High	Explicit (hospital fund)
Portugal	DRG + package model	Medium	Explicit via committee
Spain	Global budget (public sector)	Not specified	Not explicitly itemized
Sweden	DRG bundled	Medium	Hospital-managed
Switzerland	DRG bundled	High	Explicit or hospital-defined
UK	HRG + consumables-on-top	Medium	Consumables reimbursed separately

Indicative reimbursement levels are categorized as high (> €8000), medium (€5000–€8000), or low (< €5000), based on data provided by interviewees and public sources. These ranges reflect typical bundled payments (e.g. DRG tariffs) for complex ablations (e.g. left-sided AF or VT) and do not represent exact centre-level prices. Consumables coverage varies by system and may be either explicitly reimbursed or implicitly absorbed in broader procedural payments. Reimbursement values may vary significantly between centres, payers, and case complexity.

## Data Availability

The datasets generated and analysed during the current study are not publicly available due to confidentiality agreements and the qualitative nature of the data, which include personal professional opinions expressed during interviews. To protect participant anonymity, full transcripts and audio recordings cannot be shared. Summarized or aggregated data supporting the findings of this study are available from the corresponding author upon reasonable request.

## Results

*Table 1* summarizes the procurement characteristics across the 21 European countries included in this study. These include the procurement level, clinician involvement, access to innovation, evaluation methods, reimbursement mechanisms, and sustainability integration. Thematic findings from expert interviews are elaborated in the six subsections below. While the total sample comprised 22 participants, some countries were represented by more than one interviewee, whereas others were represented by a single expert (the distribution of experts per country is shown in *Figure 1*).

### Procurement models

Procurement of EP consumables varies widely across Europe. National-level procurement, characterized by centralized purchasing through ministries of health or national health insurance funds, predominates in Bulgaria, Estonia, Latvia, Lithuania, and Norway. These systems are typically highly structured and emphasize cost-efficiency.^6–10^

A second group of countries relies on regional-level procurement, where regional authorities assume purchasing responsibility. Countries employing this approach include Denmark, Finland, Italy, Spain, and Sweden. While Austria is often described as having regional-level procurement for CIEDs, EP consumables are generally procured at the hospital level through direct negotiations. No formal tenders for EP devices were reported and procurement decisions are often clinician-led and highly dependent on hospital size and budget.^11–16^

The most decentralized systems, based at the hospital level, are found in Belgium, France, Germany, the Netherlands, Poland, Portugal, Romania, Serbia, and Switzerland. Here, institutions conduct their own tenders, enabling more tailored procurement aligned with procedural requirements. However, this approach relies heavily on local procurement expertise and procedural volumes.^17–25^

It is important to note that procurement systems are not always rigid. Several countries apply hybrid approaches, adapting procurement strategies to device category, institutional capabilities, or clinical context. *Figure 2* illustrates the dominant procurement model per country based on interview responses

### Evaluation methods: cost vs. clinical criteria

The weighting of cost vs. quality and technical performance in EP procurement also varies. In price-dominant systems, such as those in Bulgaria, Estonia, Latvia, Lithuania, Poland, and Serbia, contracts are typically awarded to the lowest bidder. Clinical performance or technical differentiation plays a minimal role in decision-making.^6–8,10,22,24^

In contrast, Austria, Belgium, France, the Netherlands, and Sweden implement the “Most Economically Advantageous Tender” (MEAT) framework, incorporating structured scorecards to evaluate both pricing and technical or clinical value. These systems balance affordability with innovation and quality.^9,11,14,17,19,21,26^

Some countries place strong emphasis on clinician-led or clinically weighted evaluation. In France, for instance, clinical input may account for up to 85% of the final score. Switzerland combines clinical performance with economic and sustainability considerations and Portugal uses multidisciplinary committees to assess clinical appropriateness.

*Figure 3* displays the scoring approach by country, categorizing systems as price-driven, balanced, or clinically led.

### Clinician involvement

The role of electrophysiologists and other clinicians in procurement ranges from active leadership to peripheral consultation. In Austria, Belgium, France, Germany, the Netherlands, Poland, Portugal, Sweden, and Switzerland, clinicians are actively engaged in defining technical specifications, reviewing tenders, and, in some cases, making final decisions.

Moderate clinical involvement is found in Denmark, Finland, Norway, and Romania, where clinicians offer input on specifications or product justification but are not involved in procurement governance or scoring.

Minimal or indirect clinical participation is reported in Estonia and Lithuania, where clinician influence is limited to setting basic functional requirements.

### Reimbursement variability across EP procedures

Reimbursement levels for EP procedures vary significantly across Europe.^27–41^ Detailed country-level reimbursement data are presented in Supplementary material online, *Tables S1*–*S5*, while *Table 2* summarizes reimbursement characteristics for countries in which procedure-level reimbursement structures could be reliably synthesized and compared; countries with global budgets, non-itemized tariffs, or insufficiently granular public data are not included.

Countries like Germany and Switzerland report high reimbursement levels, often exceeding €8000 for complex procedures such as left-sided atrial fibrillation (AF) or ventricular tachycardia (VT) ablations, with Switzerland reporting over €16 000. Poland also presents high bundled reimbursements, over €16 200, though this may include consumables and infrastructure.

In contrast, France in the public sector reimburses significantly lower amounts: €2635–€7100, while in the private sector, professional fees are often separated from institutional coverage. Countries like Austria and Italy report mid-range reimbursements, typically €6000–€9500, while Belgium and the Netherlands offer between €5000 and €8600 depending on complexity.

Spain uses a global budget model that limits procedural-level transparency. Procedure-specific trends also emerge: typical flutter and SVT ablations are generally reimbursed at lower rates than complex VT or atypical flutter procedures. UK reimbursement models may separate device costs from procedural HRG tariffs, while German DRG codes often bundle all components.

Interviewees also reported substantial variation in reimbursement criteria within countries, particularly between public and private hospitals and across regions. These discrepancies underscore the absence of harmonized reimbursement policies and raise concerns regarding their impact on technology adoption, procedural equity, and clinical outcomes.

### Access to innovation

Innovation adoption in EP procurement remains inconsistent across Europe. Austria and the Netherlands report the use of dedicated innovation budgets (5 and 10%, respectively), which help support early adoption of new technologies. France employs local innovation funds at the hospital level and Spain links reimbursement to procedure-level funding, enabling flexible adoption.

Germany, Italy, Romania, and Switzerland follow a case-by-case evaluation process, often requiring detailed justification or HTA review. This may limit the speed or scale of adoption.

More restrictive models, such as those in Bulgaria, Lithuania, and Serbia, depend on central approval, with new technologies only introduced through formal tenders or ministry decisions. This often results in significant delays in adoption and limited access to advanced tools such as high-resolution mapping systems or PFA.

## Discussion

This cross-country analysis reveals substantial variability in the procurement of EP consumables across Europe. Despite the central role of these technologies in contemporary arrhythmia care, procurement systems often remain misaligned with clinical needs, innovation pathways, and broader sustainability objectives. These findings highlight persistent tensions between centralization and flexibility, cost containment, and clinical quality and underscore the growing need to integrate value-based and environmental principles into procurement policy.

### Procurement models and clinical integration

Three predominant procurement structures were identified: hospital level, regional, and national, each associated with distinct advantages and limitations. Hospital-led procurement, as observed in Germany, the Netherlands, and Switzerland, enables greater clinical customization and responsiveness to innovation. These decentralized systems allow EP teams to align purchasing decisions with procedural demands, facilitating the adoption of advanced technologies such as PFA and high-density mapping systems. However, they may offer fewer economies of scale and limited standardization, potentially contributing to inefficiencies or inequities in access between institutions.^18–20,26^

In contrast, national-level procurement systems, common in Estonia, Lithuania, and Norway, centralize purchasing through ministries of health or public insurers, enhancing administrative efficiency and price negotiation. Nevertheless, these systems may delay the integration of novel technologies due to rigid specifications and infrequent tender cycles.^6,7,9,26^ Limited formal involvement of clinical stakeholders in centralized procurement can further exacerbate this rigidity, highlighting a structural trade-off between cost control and clinical adaptability.

### Clinician involvement: a determinant of value alignment

The extent of clinician involvement emerged as a key factor influencing the alignment of procurement decisions with clinical value. In countries such as France, Belgium, Poland, and Sweden, electrophysiologists are formally engaged in drafting technical specifications and evaluating tenders.^26^ This structured involvement supports procurement decisions that better reflect real-world procedural requirements, including signal quality, anatomical compatibility, and device usability.

In contrast, many systems rely on informal or consultative clinician input that is not embedded within procurement governance. As EP procedures continue to increase in complexity, the absence of structured clinical engagement risks procurement decisions that undervalue procedural performance or safety. Embedding clinical expertise within procurement committees, particularly for technically demanding devices such as contact-force sensing catheters or visualizable sheaths, may therefore be critical to aligning purchasing decisions with patient outcomes and institutional quality standards.

### Evaluation criteria and value-based procurement

Interview findings demonstrate marked variation in evaluation criteria, particularly regarding the relative weighting of cost vs. technical or clinical performance. Price-dominant procurement frameworks may achieve short-term cost reductions but risk underestimating long-term value. In contrast, systems adopting the MEAT approach allow price considerations to be balanced with clinical evidence and technical merit.^26^

Several countries, including Finland and Italy, have moved towards value-based procurement models that incorporate lifecycle costs and outcome-oriented metrics into decision-making.^15,16,26^ These approaches support the adoption of technologies that, although potentially more expensive upfront, may deliver greater long-term efficacy, safety, and system-level efficiency. Broader integration of value-based healthcare (VBHC) principles within procurement frameworks may therefore serve as an important lever for innovation, cost-effectiveness, and sustainability.

### Role of health technology assessment in value-based procurement

Despite increasing emphasis on value-based procurement, the formal integration of health technology assessment (HTA) into EP device tendering remains limited across Europe. While some systems apply cost-effectiveness thresholds or require clinical justification for high-cost innovations, structured HTA processes are not consistently embedded in procurement governance. In countries such as Italy and Germany, HTA input may be applied on a case-by-case basis at the hospital or regional level, but this approach lacks standardization and transparency. In more centralized systems, national HTA bodies may indirectly shape procurement specifications; however, interviewees reported that these evaluations rarely extend specifically to EP consumables.

The absence of routine HTA application to EP technologies represents a missed opportunity to align procurement decisions with broader VBHC objectives. Greater integration of HTA frameworks, including comparative effectiveness, budget impact analyses, and real-world evidence, could enhance transparency, support evidence-informed investment in innovation, and promote more equitable access across regions.^42–44^

### Environmental sustainability as an emerging criterion

Environmental sustainability is increasingly recognized in medical procurement, yet formal integration remains limited. In EP, reliance on single-use catheters and extensive packaging contributes substantially to medical waste. EHRA surveys indicate high clinician awareness of environmental impact and willingness to adopt more sustainable practices, but procurement frameworks rarely include explicit environmental criterio.^2,3^

Some countries, including Sweden and the Netherlands, have begun piloting ‘green procurement’ scoring systems that incorporate lifecycle assessments and waste reduction metrics. Integrating sustainability indicators, such as device reusability, packaging volume, and carbon footprint, into tender evaluations could align procurement practices with the EU Green Deal and support a more circular economy within EP.^2,3^ Wider adoption of sustainability scoring, including lifecycle assessments and reprocessing policies, may help reconcile innovation with environmental responsibility.

## Conclusions

This qualitative, cross-country analysis demonstrates marked heterogeneity in the procurement of EP consumables across Europe. Procurement systems vary substantially in governance structure, evaluation criteria, clinician involvement, reimbursement mechanisms, and the extent to which innovation and sustainability considerations are incorporated.

Evaluation approaches range from price-dominant models to more balanced or clinically weighted frameworks, with variable alignment to value-based care principles. Similarly, clinician engagement in procurement processes differs widely, influencing how effectively purchasing decisions reflect procedural requirements and clinical priorities. Reimbursement structures and funding transparency also vary across countries and procedures, with potential implications for equitable access to advanced EP technologies.

Access to innovation remains inconsistent, shaped by differences in procurement flexibility, reimbursement pathways, and the availability of dedicated innovation funding. Environmental sustainability, while increasingly recognized as a shared concern, is rarely embedded in formal procurement criteria despite emerging initiatives in some health systems.

Taken together, these findings highlight the complexity of EP procurement across Europe and underscore the potential value of greater alignment between procurement practices, clinical needs, and broader health system objectives. Professional societies and European-level stakeholders may contribute by supporting shared principles and guidance that promote value-based, transparent, and sustainable procurement while accommodating national and regional contexts.
