# Supplementary material for: Electrophysiology consumables procurement in Europe: implications for access, innovation and value-based care
Source: Europace. 2026 Mar 10;28(5):euag039. doi: 10.1093/europace/euag039 (PMC13179736; doi:10.1093/europace/euag039)
Supplement: euag039_Supplementary_Data [file euag039_supplementary_data.zip › EP Procurement in Europe APPENDIX.docx]

Electrophysiology Consumables Procurement in Europe: Implications for Access, Innovation and Value-Based Care

Lucía Osoro^1,2,3^, Nikola Kozhuharov ^2,4^, Runa Landen ^2, 5^, Elena Arbelo ^2,6^, Martin Martinek ^7^, Christophe Leclerq ^8^, Laurent Fauchier ^9^ , Jean-Claude Deharo ^10^, Serge Boveda ^11^, Philipp Sommer ^12^, Michiel Rienstra ^13^, Piotr Szymanski ^14^, Michal Farkowski^15^, Francisco Costa ^16^, Diana Tint ^17^, Stefan Simovic ^18,19^, Krasimir Dzhinsov ^20^, Francisco Leyva ^21^, Giuseppe Boriani ^22^, Josep Figueras ^23^, Zenichi Ihara ^24^, Jose Luis Merino ^25^, Haran Burri ^26^ , Helmut Pürerfellner ^7^, Rubén Casado-Arroyo^1,2^

### **Affiliations**

^1^ Department of Cardiology, H.U.B.-Hôpital Erasme, Université Libre de Bruxelles, Brussels 1070, Belgium.

^2^ EHRA Advocacy, Quality Improvement, and Health Economics Committee (European Heart Rhythm Association).

3 Centro Universitario HM Hospitales de Ciencias de la Salud (CUHMED), Universidad Camilo José Cela. Madrid, Spain

4 University Hospital Bern - Inselspital, Freiburgstrasse 20, 3010 Bern, Switzerland

5 Institute of Medicine - Sahlgrenska Academy - University of Gothenburg, Gothenburg , Sweden

6 Department of Cardiology, Hospital Clinic, Barcelona, Spain

7 Ordensklinikum Linz Elisabethinen, Linz , Austria

8 CHU Rennes - Hôpital Pontchaillou, France

9 Hôpital Trousseau, CHRU de Tours, France

10 Assistance Publique − Hôpitaux de Marseille, Centre Hospitalier Universitaire La Timone, Service de Cardiologie, Marseille, France and Aix Marseille Université, C2VN, Marseille, France

11 Clinique Pasteur, Toulouse, France

12 Heart and Diabetes Center North Rhine-Westphalia, University clinic of Bochum, Bad Oeynhausen, Germany

13 University of Groningen, University Medical Centre Groningen, Groningen, The Netherlands

14 Centre of Postgraduate Medical Education, Warsaw, Poland

15 Ministry of Interior and Administration National Medical Institute, Warsaw, Poland

16 Hospital da Luz, SA, Lisbon, Portugal

17 Transilvania University of Brasov, Brasov, Romania

18 Department of Internal Medicine, Faculty of Medical Sciences, University of Kragujevac, Kragujevac Serbia

19 Clinic for Cardiology, University Clinical Centre Kragujevac, Kragujevac, Serbia

20  University Hospital "Sveti Georgi", Plovdiv, Bulgaria

21 Aston University, Birmingham, United Kingdom of Great Britain & Northern Ireland

22 Policlinico di Modena, Italy

23 European Health Observatory, Brussels, Belgium

24 Health Economics and Reimbursement, Abbott, Zaventem, Belgium

25 La Paz University Hospital, Madrid (Spain)

26 Cardiology Department of the University Hospital of Geneva , Switzerland

**Corresponding author:**

Department of Cardiology, H.U.B.-Hôpital Erasme, Université Libre de Bruxelles, Brussels 1070, Belgium.

Email: [ruben.casadoarroyo@hubruxelles.be](mailto:ruben.casadoarroyo@hubruxelles.be)

Word Count: 3299

**Appendix**

| Participant ID | Country | Role | Institution | Experience Summary |
| --- | --- | --- | --- | --- |
| P1 | Austria | Cardiologist | Ordensklinikum Linz Elisabethinen | Direct involvement in CIED procurement or implantation |
| P2 | Belgium | Cardiologist | Hospital Êrasme of Bruxelles | Direct involvement in CIED procurement or implantation |
| P3 | France | Cardiologist | Hospital Pontchaillou of Rennes | Direct involvement in CIED procurement or implantation |
| P4 | France | Cardiologist | Hôpital Trousseau, CHRU de Tours | Direct involvement in CIED procurement or implantation |
| P5 | France | Cardiologist | Assistance Publique − Hôpitaux de Marseille, Centre Hospitalier Universitaire La Timone | Direct involvement in CIED procurement or implantation |
| P6 | France | Cardiologist | Clinique Pasteur, Toulouse | Direct involvement in CIED procurement or implantation |
| P7 | Germany | Cardiologist | Heart and Diabetes Center NRW, Bad Oeynhausen | Direct involvement in CIED procurement or implantation |
| P8 | Italy | Cardiologist | Modena Polyclinic Modena University Hospita | Direct involvement in CIED procurement or implantation |
| P09 | Netherlands | Cardiologist | University of Groningen, University Medical Centre Groningen | Direct involvement in CIED procurement or implantation |
| P10 | Poland | Cardiologist | National Institute of Medicine MSWiA | Direct involvement in CIED procurement or implantation |
| P11 | Poland | Cardiologist | Ministry of Interior and Administration National Medical Institute | Direct involvement in CIED procurement or implantation |
| P12 | Portugal | Cardiologist | Hospital da Luz, SA, Lisbon | Direct involvement in CIED procurement or implantation |
| P13 | Romania | Cardiologist | Transilvania University of Brasov | Direct involvement in CIED procurement or implantation |
| P14 | Spain | Cardiologist | Hospital Universitario La Paz | Direct involvement in CIED procurement or implantation |
| P15 | Spain | Cardiologist | Hospital Clinic | Direct involvement in CIED procurement or implantation |
| P16 | Sweden | Cardiologist | Institute of Medicine - Sahlgrenska Academy | Direct involvement in CIED procurement or implantation |
| P17 | Switzerland | Cardiologist | Geneva University Hospitals | Direct involvement in CIED procurement or implantation |
| P18 | Switzerland | Cardiologist | Bern University Hospital | Direct involvement in CIED procurement or implantation |
| P19 | Serbia | Cardiologist | Faculty of Medical Sciences, University of Kragujevac | Direct involvement in CIED procurement or implantation |
| P20 | Bulgaria | Cardiologist | University Hospital "Sveti Georgi" | Direct involvement in CIED procurement or implantation |
| P21 | UK | Cardiologist | Aston University, Birmingham | Direct involvement in CIED procurement or implantation |
| P22 | Baltic & Nordic | Industry representative |  | Direct involvement in CIED procurement and sales processes |
