# Supplementary material for: Electrophysiology consumables procurement in Europe: implications for access, innovation and value-based care
Source: Europace. 2026 Mar 10;28(5):euag039. doi: 10.1093/europace/euag039 (PMC13179736; doi:10.1093/europace/euag039)
Supplement: euag039_Supplementary_Data [file euag039_supplementary_data.zip › EP Procurement in Europe SUPPLEMENTS.docx]

Electrophysiology Consumables Procurement in Europe: Implications for Access, Innovation and Value-Based Care

Lucía Osoro^1,2,3^, Nikola Kozhuharov ^2,4^, Runa Landen ^2, 5^, Elena Arbelo ^2,6^, Martin Martinek ^7^, Christophe Leclerq ^8^, Laurent Fauchier ^9^ , Jean-Claude Deharo ^10^, Serge Boveda ^11^, Philipp Sommer ^12^, Michiel Rienstra ^13^, Piotr Szymanski ^14^, Michal Farkowski^15^, Francisco Costa ^16^, Diana Tint ^17^, Stefan Simovic ^18,19^, Krasimir Dzhinsov ^20^, Francisco Leyva ^21^, Giuseppe Boriani ^22^, Josep Figueras ^23^, Zenichi Ihara ^24^, Jose Luis Merino ^25^, Haran Burri ^26^ , Helmut Pürerfellner ^7^, Rubén Casado-Arroyo^1,2^

### **Affiliations**

^1^ Department of Cardiology, H.U.B.-Hôpital Erasme, Université Libre de Bruxelles, Brussels 1070, Belgium.

^2^ EHRA Advocacy, Quality Improvement, and Health Economics Committee (European Heart Rhythm Association).

3 Centro Universitario HM Hospitales de Ciencias de la Salud (CUHMED), Universidad Camilo José Cela. Madrid, Spain

4 University Hospital Bern - Inselspital, Freiburgstrasse 20, 3010 Bern, Switzerland

5 Institute of Medicine - Sahlgrenska Academy - University of Gothenburg, Gothenburg , Sweden

6 Department of Cardiology, Hospital Clinic, Barcelona, Spain

7 Ordensklinikum Linz Elisabethinen, Linz , Austria

8 CHU Rennes - Hôpital Pontchaillou, France

9 Hôpital Trousseau, CHRU de Tours, France

10 Assistance Publique − Hôpitaux de Marseille, Centre Hospitalier Universitaire La Timone, Service de Cardiologie, Marseille, France and Aix Marseille Université, C2VN, Marseille, France

11 Clinique Pasteur, Toulouse, France

12 Heart and Diabetes Center North Rhine-Westphalia, University clinic of Bochum, Bad Oeynhausen, Germany

13 University of Groningen, University Medical Centre Groningen, Groningen, The Netherlands

14 Centre of Postgraduate Medical Education, Warsaw, Poland

15 Ministry of Interior and Administration National Medical Institute, Warsaw, Poland

16 Hospital da Luz, SA, Lisbon, Portugal

17 Transilvania University of Brasov, Brasov, Romania

18 Department of Internal Medicine, Faculty of Medical Sciences, University of Kragujevac, Kragujevac Serbia

19 Clinic for Cardiology, University Clinical Centre Kragujevac, Kragujevac, Serbia

20  University Hospital "Sveti Georgi", Plovdiv, Bulgaria

21 Aston University, Birmingham, United Kingdom of Great Britain & Northern Ireland

22 Policlinico di Modena, Italy

23 European Health Observatory, Brussels, Belgium

24 Health Economics and Reimbursement, Abbott, Zaventem, Belgium

25 La Paz University Hospital, Madrid (Spain)

26 Cardiology Department of the University Hospital of Geneva , Switzerland

**Corresponding author:**

Department of Cardiology, H.U.B.-Hôpital Erasme, Université Libre de Bruxelles, Brussels 1070, Belgium.

Email: [ruben.casadoarroyo@hubruxelles.be](mailto:ruben.casadoarroyo@hubruxelles.be)

Word Count: 3299

# Supplementary Table S1-S6

| Inpatient | Atrial Fibrillation Ablation | | | | |
| --- | --- | --- | --- | --- | --- |
| Country | **Physician Fee** | **Procedure** | **Device** | **Total (or DRG)** | **Reimbursement source** |
| Germany (31) |  | 4.699,16 € | 4.067,31 € | 8.766,47 € (1) | G-DRG |
| United Kingdom (30) |  |  | Paid on top | 5.898,58 € (2) | NHS England |
| France (public) (29) |  |  |  | 7.099,21 € (3) | Assurance Maladie |
| France (private)(29) | 1.545,01 € |  |  | 6.594,73 € (4) | Assurance Maladie |
| Italy (33) |  |  |  | 6.980,00 € (5) | Italian MoH |
| Spain (35) | - | - | - | - (6) | Servicio Nacional de Salud |
| Switzerland (36) |  | 8.217,89 € | 8.677,45 € | 16.895,34 € (7) | Swiss DRG |
| Austria (27) |  | 8.457,00 € |  | 9.574,00 € (8) | Austria MoH |
| Belgium (28) |  | 2.932,61 € | 3.293,10 € | 6.225,71 € (9) | INAMI |
| The Netherlands (37) |  |  |  | 11.928,00 € (10) | Zorginstituut Nederland |
| Poland (34) |  |  |  | 16.207,86 € (11) | NFZ Poland |
| Denmark (38) |  |  |  | 7.938,72 € (12) | \|  \| \| --- \|  \| Danish Medicines Agency (DKMA) \| \| --- \| |
| Sweden (40) |  |  |  | 11.832,99 € (13) | TLV |
| Norway (39) |  |  |  | 7.528,14 € (14) | Norwegian Medical Products Agency (NoMA) |
| Czech Republic (41) |  |  |  | 9.695,53 € (15) | \|  \| \| --- \|  \| State Institute for Drug Control (SÚKL) \| \| --- \| |

Supplementary Table 1: Reimbursement pricing per country for Atrial Fibrillation ablation

1. F50A covers left sided ablation irrespective of complexity
2. AF PVI leads to complex ablation HRG where the catheters are paid on top of the tariff
3. CCAM procedure code DEPF033 falls into 05K191 in 90% of its cases.
4. CCAM procedure code DEPF033 falls into 05K191 in 92.4% of its cases.
5. Lombardia regional DRG 555 & 518 covers AF
6. No reimbursement. Global budget based country
7. F50A covers left sided ablation irrespective of complexity
8. LKF to EUR point conversion assumed 1:1. MEL21.04 A covers PVI irrespective of complexity
9. Assumed to include 3D mapping on top of ablation catheters
10. Class 4 ablation. Tariff shows without overnight stay. With overnight stay, tariff is € 14,419. Most commonly done for AF
11. AF PVI leads to DRG E48
12. Tariff for ablation with mapping. Without mapping, tariff is € 4,936
13. Tariff for complex ablation. Day case tariff is € 6,404
14. Tariff for ablation without overnight stay. With overnight stay, tariff is € 8,946
15. Tariff for complex ablation. Simple ablation tariff is € 5,472. AV junction ablation tariff is € 3,880

| Inpatient | Atrial Flutter Typical Right Sided Ablation | | | | |
| --- | --- | --- | --- | --- | --- |
| Country | **Physician Fee** | **Procedure** | **Device** | **Total (or DRG)** | **Reimbursement source** |
| Germany (31) |  | 4.065,15 € | 3.044,70 € | 7.109,85 € (1) | G-DRG |
| United Kingdom (30) |  |  |  | 3.186,55 € (2) | NHS England |
| France (public) (29) |  |  |  | 2.634,62 € (3) | Assurance Maladie |
| France (private)(29) | 472,54 € |  |  | 2.397,75 € (4) | Assurance Maladie |
| Italy (33) |  |  |  | 6.041,00 € (5) | Italian MoH |
| Spain (35) | - | - | - | - (6) | Servicio Nacional de Salud |
| Switzerland (36) |  | 5.997,70 € | 6.987,14 € | 12.984,84 € (7) | Swiss DRG |
| Austria (27) |  | 4.824,00 € |  | 5.868,00 € (8) | Austria MoH |
| Belgium (28) |  | 1.789,27 € | 3.293,10 € | 5.082,37 € (9) | INAMI |
| The Netherlands (37) |  |  |  | 8.619,00 € (10) | Zorginstituut Nederland |
| Poland (34) |  |  |  | 16.713,80 € (11) | NFZ Poland |
| Denmark (38) |  |  |  | 7.938,72 € (12) | \|  \| \| --- \|  \| Danish Medicines Agency (DKMA) \| \| --- \| |
| Sweden (40) |  |  |  | 6.404,42 € (13) | TLV |
| Norway (39) |  |  |  | 7.528,14 € (14) | Norwegian Medical Products Agency (NoMA) |
| Czech Republic (41) |  |  |  | 5.472,06 € (15) | \|  \| \| --- \|  \| State Institute for Drug Control (SÚKL) \| \| --- \| |

Supplementary Table 2: Reimbursement per country for Atrial Flutter ablation in the right side

1. F50A covers atypical left sided aflutter and F50B covers typical right sided aflutter (incl HDG otherwise F50C)
2. VT, PVI, and 3D mapping combination would lead to the complex ablation
3. CCAM procedure code DEPF014 for left sided falls into 05K191 in 83.2% of cases and DEPF012 for right sided falls into 05K19 in 29% of cases and 54% of cases into 05K20T/1
4. CCAM procedure code DEPF014 for left sided falls into 05K191 in 92% of cases and DEPF012 for right sided falls into 05K19 in 29% of cases and 53% of cases into 05K20T/1
5. Lombardia regional DRG 518 aflutter falls under other cardiac arrhythmias
6. No reimbursement. Global budget based country
7. Left sided ablation automatically maps into F50A, right sided leads to F50B (incl HDG otherwise F50C)
8. LKF to EUR point conversion assumed 1:1. MEL21.04 B covers all ablations except ventricular and PVI, otherwise MEL21.04 A
9. Assumed to include 3D mapping on top of ablation catheters
10. Class 2 ablation. Tariff shows without overnight stay. With overnight stay, tariff is €10,563. Aflutter & SVT most common diagnoses
11. Left sided aflutter leads to E48 and right sided aflutter leads to E47 for complex 3D mapping ablation
12. Tariff for ablation with mapping. Without mapping, tariff is € 4,936
13. Tariff without overnight stay. With overnight stay, simple ablation is € 10,491 or € 11,832 for complex
14. Tariff for ablation without overnight stay. With overnight stay, tariff is € 8,946
15. Tariff for simple ablation. Complex ablation tariff is € 9,695. AV junction ablation tariff is € 3,880

| Inpatient | VT Left Sided Ablation | | | | |
| --- | --- | --- | --- | --- | --- |
| Country | **Physician Fee** | **Procedure** | **Device** | **Total (or DRG)** | **Reimbursement source** |
| Germany (31) |  | 4.699,16 € | 4.067,31 € | 8.766,47 € (1) | G-DRG |
| United Kingdom (30) |  |  | Paid on top | 5.898,58 € (2) | NHS England |
| France (public) (29) |  |  |  | 2.634,62 € (3) | Assurance Maladie |
| France (private)(29) | 418,00 € |  |  | 2.397,75 € (4) | Assurance Maladie |
| Italy (33) |  |  |  | 6.980,00 € (5) | Italian MoH |
| Spain (35) | - | - | - | - (6) | Servicio Nacional de Salud |
| Switzerland (36) |  | 8.217,89 € | 8.677,45 € | 16.895,34 € (7) | Swiss DRG |
| Austria (27) |  | 8.457,00 € |  | 9.574,00 € (8) | Austria MoH |
| Belgium (28) |  | 1.960,97 € | 3.293,10 € | 5.254,07 € (9) | INAMI |
| The Netherlands (37) |  |  |  | 8.546,00 € (10) | Zorginstituut Nederland |
| Poland (34) |  |  |  | 16.713,80 € (11) | NFZ Poland |
| Denmark (38) |  |  |  | 7.938,72 € (12) | \|  \| \| --- \|  \| Danish Medicines Agency (DKMA) \| \| --- \| |
| Sweden (40) |  |  |  | 6.404,42 € (13) | TLV |
| Norway (39) |  |  |  | 7.528,14 € (14) | Norwegian Medical Products Agency (NoMA) |
| Czech Republic (41) |  |  |  | 5.472,06 € (15) | \|  \| \| --- \|  \| State Institute for Drug Control (SÚKL) \| \| --- \| |

Supplementary Table 3: Reimbursement for Ventricular Tachycardia ablation in the left side

1. F50A covers atypical left sided aflutter and F50B covers typical right sided aflutter (incl HDG otherwise F50C)
2. VT, PVI, and 3D mapping combination would lead to the complex ablation
3. CCAM procedure code DEPF014 for left sided falls into 05K191 in 83.2% of cases and DEPF012 for right sided falls into 05K19 in 29% of cases and 54% of cases into 05K20T/1
4. CCAM procedure code DEPF014 for left sided falls into 05K191 in 92% of cases and DEPF012 for right sided falls into 05K19 in 29% of cases and 53% of cases into 05K20T/1
5. Lombardia regional DRG 518 aflutter falls under other cardiac arrhythmias
6. No reimbursement. Global budget based country
7. Left sided ablation automatically maps into F50A, right sided leads to F50B (incl HDG otherwise F50C)
8. LKF to EUR point conversion assumed 1:1. MEL21.04 B covers all ablations except ventricular and PVI, otherwise MEL21.04 A
9. Assumed to include 3D mapping on top of ablation catheters
10. Class 2 ablation. Tariff shows without overnight stay. With overnight stay, tariff is €10,563. Aflutter & SVT most common diagnoses
11. Left sided aflutter leads to E48 and right sided aflutter leads to E47 for complex 3D mapping ablation
12. Tariff for ablation with mapping. Without mapping, tariff is € 4,936
13. Tariff without overnight stay. With overnight stay, simple ablation is € 10,491 or € 11,832 for complex
14. Tariff for ablation without overnight stay. With overnight stay, tariff is € 8,946
15. Tariff for simple ablation. Complex ablation tariff is € 9,695

| Inpatient | VT Right Sided Ablation | | | |  |
| --- | --- | --- | --- | --- | --- |
| Country | **Physician Fee** | **Procedure** | **Device** | **Total (or DRG)** | **Reimbursement source** |
| Germany (31) |  | 4.065,15 € | 3.044,70 € | 7.109,85 € (1) | G-DRG |
| United Kingdom (30) |  |  | Paid on top | 5.898,58 € (2) | NHS England |
| France (public) (29) |  |  |  | 2.634,62 € (3) | Assurance Maladie |
| France (private)(29) | 418,00 € |  |  | 2.397,75 € (4) | Assurance Maladie |
| Italy (33) |  |  |  | 6.980,00 € (5) | Italian MoH |
| Spain (35) | - | - | - | - (6) | Servicio Nacional de Salud |
| Switzerland (36) |  | 5.997,70 € | 6.987,14 € | 12.984,84 € (7) | Swiss DRG |
| Austria (27) |  | 8.457,00 € |  | 9.574,00 € (8) | Austria MoH |
| Belgium (28) |  | 1.960,97 € | 3.293,10 € | 5.254,07 € (9) | INAMI |
| The Netherlands (37) |  |  |  | 8.546,00 € (10) | Zorginstituut Nederland |
| Poland (34) |  |  |  | 16.713,80 € (11) | NFZ Poland |
| Denmark (38) |  |  |  | 7.938,72€ (12) | \|  \| \| --- \|  \| Danish Medicines Agency (DKMA) \| \| --- \| |
| Sweden (40) |  |  |  | 6.404,42 € (13) | TLV |
| Norway (39) |  |  |  | 7.528,14 € (14) | Norwegian Medical Products Agency (NoMA) |
| Czech Republic (41) |  |  |  | 5.472,06 € (15) | \|  \| \| --- \|   State Institute for Drug Control (SÚKL) |

Supplementary Table 4: Reimbursement of Ventricular Tachycardia ablation in the right side

1. F50A covers atypical left sided aflutter and F50B covers typical right sided aflutter (incl HDG otherwise F50C)
2. VT, PVI, and 3D mapping combination would lead to the complex ablation
3. CCAM procedure code DEPF014 for left sided falls into 05K191 in 83.2% of cases and DEPF012 for right sided falls into 05K19 in 29% of cases and 54% of cases into 05K20T/1
4. CCAM procedure code DEPF014 for left sided falls into 05K191 in 92% of cases and DEPF012 for right sided falls into 05K19 in 29% of cases and 53% of cases into 05K20T/1
5. Lombardia regional DRG 518 aflutter falls under other cardiac arrhythmias
6. No reimbursement. Global budget based country
7. Left sided ablation automatically maps into F50A, right sided leads to F50B (incl HDG otherwise F50C)
8. LKF to EUR point conversion assumed 1:1. MEL21.04 B covers all ablations except ventricular and PVI, otherwise MEL21.04 A
9. Assumed to include 3D mapping on top of ablation catheters
10. Class 2 ablation. Tariff shows without overnight stay. With overnight stay, tariff is €10,563. Aflutter & SVT most common diagnoses
11. Left sided aflutter leads to E48 and right sided aflutter leads to E47 for complex 3D mapping ablation
12. Tariff for ablation with mapping. Without mapping, tariff is € 4,936
13. Tariff without overnight stay. With overnight stay, simple ablation is € 10,491 or € 11,832 for complex
14. Tariff for ablation without overnight stay. With overnight stay, tariff is € 8,946
15. Tariff for simple ablation. Complex ablation tariff is € 9,695

| Inpatient | SVT Left Sided Ablation | | | | |
| --- | --- | --- | --- | --- | --- |
| Country | **Physician Fee** | **Procedure** | **Device** | **Total (or DRG)** | **Reimbursement source** |
| Germany (31) |  | 4.699,16 € | 4.067,31 € | 8.766,47 € (1) | G-DRG |
| United Kingdom (30) |  |  |  | 3.186,55 € (2) | NHS England |
| France (public) (29) |  |  |  | 2.634,62 € (3) | Assurance Maladie |
| France (private)(29) | 654,37 € |  |  | 2.397,75 € (4) | Assurance Maladie |
| Italy (33) |  |  |  | 6.041,00 € (5) | Italian MoH |
| Spain (35) | - | - | - | - (6) | Servicio Nacional de Salud |
| Switzerland (36) |  | 8.217,89 € | 8.677,45 € | 16.895,34 € (7) | Swiss DRG |
| Austria (27) |  | 4.824,00 € |  | 5.868,00 € (8) | Austria MoH |
| Belgium (28) |  | 1.635,28 € | 3.293,10 € | 4.928,38 € (9) | INAMI |
| The Netherlands (37) |  |  |  | 8.619,00 € (10) | Zorginstituut Nederland |
| Poland (34) |  |  |  | 9.846,02 € (11) | NFZ Poland |
| Denmark (38) |  |  |  | 7.938,72 € (12) | \|  \| \| --- \|   Danish Medicines Agency (DKMA) |
| Sweden (40) |  |  |  | 6.404,42 € (13) | TLV |
| Norway (39) |  |  |  | 7.528,14 € (14) | Norwegian Medical Products Agency (NoMA) |
| Czech Republic (41) |  |  |  | 3.880,89 € (15) | \|  \| \| --- \|   State Institute for Drug Control (SÚKL) |

Supplementary Table 5: Supraventricular tachycardia ablation in the left side

1. F50A covers atypical left sided aflutter and F50B covers typical right sided aflutter (incl HDG otherwise F50C)
2. VT, PVI, and 3D mapping combination would lead to the complex ablation
3. CCAM procedure code DEPF014 for left sided falls into 05K191 in 83.2% of cases and DEPF012 for right sided falls into 05K19 in 29% of cases and 54% of cases into 05K20T/1
4. CCAM procedure code DEPF014 for left sided falls into 05K191 in 92% of cases and DEPF012 for right sided falls into 05K19 in 29% of cases and 53% of cases into 05K20T/1
5. Lombardia regional DRG 518 aflutter falls under other cardiac arrhythmias
6. No reimbursement. Global budget based country
7. Left sided ablation automatically maps into F50A, right sided leads to F50B (incl HDG otherwise F50C)
8. LKF to EUR point conversion assumed 1:1. MEL21.04 B covers all ablations except ventricular and PVI, otherwise MEL21.04 A
9. Assumed to include 3D mapping on top of ablation catheters
10. Class 2 ablation. Tariff shows without overnight stay. With overnight stay, tariff is €10,563. Aflutter & SVT most common diagnoses
11. Left sided aflutter leads to E48 and right sided aflutter leads to E47 for complex 3D mapping ablation
12. Tariff for ablation with mapping. Without mapping, tariff is € 4,936
13. Tariff without overnight stay. With overnight stay, simple ablation is € 10,491 or € 11,832 for complex
14. Tariff for ablation without overnight stay. With overnight stay, tariff is € 8,946
15. Tariff for AV junction ablation. Complex ablation tariff is € 9,695. Simple ablation tariff is € 5,472

| Inpatient | SVT Right Sided Ablation | | | | |
| --- | --- | --- | --- | --- | --- |
| Country | **Physician Fee** | **Procedure** | **Device** | **Total (or DRG)** | **Reimbursement source** |
| Germany (31) |  | 4.065,15 € | 3.044,70 € | 7.109,85 € (1) | G-DRG |
| United Kingdom (30) |  |  |  | 3.186,55 € (2) | NHS England |
| France (public) (29) |  |  |  | 2.634,62 € (3) | Assurance Maladie |
| France (private)(29) | 654,37 € |  |  | 2.397,75 € (4) | Assurance Maladie |
| Italy (33) |  |  |  | 6.041,00 € (5) | Italian MoH |
| Spain (35) | - | - | - | - (6) | Servicio Nacional de Salud |
| Switzerland (36) |  | 5.997,70 € | 6.987,14 € | 12.984,84 € (7) | Swiss DRG |
| Austria (27) |  | 4.824,00 € |  | 5.868,00 € (8) | Austria MoH |
| Belgium (28) |  | 1.635,28 € | 3.293,10 € | 4.928,38 € (9) | INAMI |
| The Netherlands (37) |  |  |  | 8.619,00 € (10) | Zorginstituut Nederland |
| Poland (34) |  |  |  | 9.846,02 € (11) | NFZ Poland |
| Denmark (38) |  |  |  | 7.938,72 € (12) | \|  \| \| --- \|   Danish Medicines Agency (DKMA) |
| Sweden (40) |  |  |  | 6.404,42 € (13) | TLV |
| Norway (39) |  |  |  | 7.528,14 € (14) | Norwegian Medical Products Agency (NoMA) |
| Czech Republic (41) |  |  |  | 3.880,89 € (15) | \|  \| \| --- \|   State Institute for Drug Control (SÚKL) |

Supplementary Table 6: Reimbursement for Supraventricular Tachycardia ablation in the right side

1. F50A covers atypical left sided aflutter and F50B covers typical right sided aflutter (incl HDG otherwise F50C)
2. VT, PVI, and 3D mapping combination would lead to the complex ablation
3. CCAM procedure code DEPF014 for left sided falls into 05K191 in 83.2% of cases and DEPF012 for right sided falls into 05K19 in 29% of cases and 54% of cases into 05K20T/1
4. CCAM procedure code DEPF014 for left sided falls into 05K191 in 92% of cases and DEPF012 for right sided falls into 05K19 in 29% of cases and 53% of cases into 05K20T/1
5. Lombardia regional DRG 518 aflutter falls under other cardiac arrhythmias
6. No reimbursement. Global budget based country
7. Left sided ablation automatically maps into F50A, right sided leads to F50B (incl HDG otherwise F50C)
8. LKF to EUR point conversion assumed 1:1. MEL21.04 B covers all ablations except ventricular and PVI, otherwise MEL21.04 A
9. Assumed to include 3D mapping on top of ablation catheters
10. Class 2 ablation. Tariff shows without overnight stay. With overnight stay, tariff is €10,563. Aflutter & SVT most common diagnoses
11. Left sided aflutter leads to E48 and right sided aflutter leads to E47 for complex 3D mapping ablation
12. Tariff for ablation with mapping. Without mapping, tariff is € 4,936
13. Tariff without overnight stay. With overnight stay, simple ablation is € 10,491 or € 11,832 for complex
14. Tariff for ablation without overnight stay. With overnight stay, tariff is € 8,946
15. Tariff for AV junction ablation. Complex ablation tariff is € 9,695. Simple ablation tariff is € 5,472

# Supplement

## S7 – Interview Guide used in the study

The following guide was used for the semi-structured interviews conducted with the participants.

- At what level is procurement of EP consumables typically conducted in your country (national, regional, hospital)?
- Is procurement centralized, or do hospitals have autonomy in decision-making?
- What are the key evaluation criteria used in EP procurement (e.g., price, quality, MEAT)?
- Are there scoring systems in place to assess tenders?
- Are electrophysiologists or cardiologists involved in the procurement process?
- To what extent can clinicians influence final purchasing decisions?
- How is the adoption of new or innovative EP devices handled in your system?
- Are there specific pathways or budgets to trial or adopt new technologies?
- How are EP procedures reimbursed in your country?
- Do rebate or volume-based discount mechanisms exist for EP consumables?
